# Infants and Air

**Published:** 1860-05

**Authors:** 


### INFANTS AND AIR.

Parliamentary returns show that of twenty-eight hundred infants annually sent to various hospitals to be taken care of, twenty-four out of twenty-five died before they were a year old! A law was immediately passed that they should be sent to the country thereafter, when it was found that only nine out of twenty-five died the first year; that is, instead of twenty-six hundred and ninety dying, there were only four hundred and fifty, a difference of twenty-two hundred and forty.

This simple unvarnished statement of an indisputable fact, ought to impress the mind of every parent deeply, with the importance and the duty of using all practicable means for securing to children the habitual breathing of the purest air possible: being careful to avoid a radical, mischievous, and most prevalent error that warm air is necessarily impure. Warmth is as essential to infantile health as pure air. How best to secure both, should be our constant study. There are more deaths under five years of age, in New-York, than there are from five to sixty years, owing to three things, a want of pure air, of suitable warmth, and proper food. In these three wants are found the overwhelming majority of causes for the fearful statement above named. Let every parent in city or country, in hovel or mansion, mature these things.

To die childless, after having been once blessed with dear children, must be one of the most terrible of all calamities of the heart; yet, in countless multitudes of cases, the sufferers are the authors of their own crushing sorrows, by reason of their unpardonable ignorance or more criminal neglect.

